# Investigation on Indoor Air Pollution and Childhood Allergies in Households in Six Chinese Cities by Subjective Survey and Field Measurements

**DOI:** 10.3390/ijerph14090979

**Published:** 2017-08-29

**Authors:** Jinhua Hu, Nianping Li, Yang Lv, Jing Liu, Jingchao Xie, Huibo Zhang

**Affiliations:** 1 College of Civil Engineering, Hunan University, Changsha 410082, China; hujinhua@hnu.edu.cn; 2 College of Civil Engineering, Dalian University of Technology, Dalian 116024, China; lvyang_dlut@163.com; 3 School of Municipal and Environmental Engineering, Harbin Institute of Technology, Harbin 150001, China; liujinghit0@163.com; 4 College of Architecture and Civil Engineering, Beijing University of Technology, Beijing 100124, China; xiejc@bjut.edu.cn; 5 School of Naval Architecture, Ocean & Civil Engineering, Shanghai Jiao Tong University, Shanghai 200240, China; zhanghuibo@sjtu.edu.cn

**Keywords:** indoor pollution, schoolchildren, allergic diseases, field investigation, fine particulate matter (PM_2.5_), semi-volatile organic compounds (SVOCs), fungi

## Abstract

Greater attention is currently being paid to the relationship between indoor environment and childhood allergies, however, the lack of reliable data and the disparity among different areas hinders reliable assessment of the relationship. This study focuses on the effect of indoor pollution on Chinese schoolchildren and the relationship between specific household and health problems suffered. The epidemiological questionnaire survey and the field measurement of the indoor thermal environment and primary air pollutants including CO_2_, fine particulate matter (PM_2.5_), chemical pollutants and fungi were performed in six Chinese cities. A total of 912 questionnaires were eligible for statistical analyses and sixty houses with schoolchildren aged 9–12 were selected for field investigation. Compared with Chinese national standards, inappropriate indoor relative humidity (<30% or >70%), CO_2_ concentration exceeding 1000 ppm and high PM_2.5_ levels were found in some monitored houses. Di(2-ethylhexyl) phthalate (DEHP) and dibutyl phthalate (DBP) were the most frequently detected semi-volatile organic compounds (SVOCs) in house dust. *Cladosporium*, *Aspergillus* and *Penicillium* were detected in both indoor air and house dust. This study indicates that a thermal environment with CO_2_ exceeding 1000 ppm, DEHP and DBP exceeding 1000 μg/g, and high level of PM_2.5_, *Cladosporium*, *Aspergillus* and *Penicillium* increases the risk of children’s allergies.

## 1. Introduction

The indoor environment is of great importance to human health as in modern society people spend approximately 90% of their time indoors, especially at their own homes [1,2,3]. In general, children spend more time indoors than adults. In such a condition, and aligned with the accelerated growth of their more immature organs which achieve higher metabolic rates [4,5], children are more susceptible to the effects of indoor environmental exposure. With the rapid modernization and urbanization of China, the prevalence of childhood allergic diseases such as asthma, rhinitis and eczema have correspondingly been rapidly increasing over the recent decades [6,7]. A large-scale survey on childhood asthma in China conducted by the National Cooperative Group indicated that the prevalence of asthma between the ages of 0–14, increased from 1.5% in 2000 to 3.02% in 2010, hence an increase of 101.3% [8,9]. Of further interest and importance in this area is the emerging and accumulating evidence indicating that both genetic and environmental factors play important roles in the etiology of allergic diseases. Results suggest that genetic factors are not the only ones for the rapid and increasingly common manifestations of allergic diseases,which makes it feasible that environmental factors are important contributors.

A growing number of studies suggest that dwelling environment, lifestyles and indoor air pollution contribute to the development of allergic disease [10]. Recent meta-analysis [11,12] concluded that living or attending schools near high traffic density roads with higher levels of motor vehicle air pollutants increased the incidence and prevalence of childhood asthma. Sun et al. [13] declared that the lifestyle and home environment exposure undergone by children between 1–8 years are important asthma and allergy risk factors in Northeast Texas. Likewise, indoor dampness is considered a risk factor for children’s health. Bornehag et al. [14] found that indoor moisture characterization increased the risk of respiratory symptoms (cough, wheezing and asthma) for children aged 1–6. In addition, a series of studies have concluded that high indoor humidity is strongly related to childhood bronchial obstruction, rhinitis and respiratory symptoms [15,16,17,18].

Carbon dioxide (CO_2_) is a natural product of human metabolism, but high CO_2_ levels can result in unhealthy symptoms such as sore throat, irritated nose/sinus, combined mucous membrane, tight chest and wheezing [19]. Indoor chemical pollutants are mainly emitted from building materials. Some evidence has clearly indicated that the increasing risk of allergy, asthma and airway inflammation in children were due to exposure to formaldehyde (HCHO) [20,21]. Additionally, volatile organic compound (VOC) emissions from building materials were related to asthma, wheezing and allergies in children [22]. Even at low concentrations of the pollutant benzene, an essential VOC category, has been proven to have an association with childhood respiratory health [23]. Fine particulate matter (PM_2.5_) is one of the major environmental concerns in China due to the prevalence of haze. Its indoor exposure could result in the increase of cumulative incidence of the lower respiratory symptoms, and also be related to allergic inflammation and persistent asthma in children [24,25]. As an indoor biological pollutant, fungi are ubiquitous in children’s indoor environments, with exposure to fungal spores being a widely recognized inducing factor as regards respiratory diseases and atopic dermatitis [26,27,28]. Additionally, fungi have been reported as a positive association of symptoms of respiratory and allergic diseases such as wheezes, daytime breathlessness, allergic rhinitis, hypersensitivity pneumonitis and doctor-diagnosed asthma [29,30]. *Cladosporium* is a significant allergen and has an adverse effect on asthmatics or respiratory diseases [31]. *Penicillium* is known to produce mycotoxins that can affect human health [32]. House dust includes mites, pollen, mold and semi-volatile organic compounds (SVOCs). The increasing awareness, in recent years about the effect of such as the above, has alerted the effect of the exposure to SVOCs in house dust on childhood asthma or allergies. It has been suggested that children are more vulnerable than adults, to phthalate exposure through household floor dust, and, in addition, it was becoming obvious that the levels of SVOCs in house dust were, infact, associated with the prevalence of allergic rhinitis, conjunctivitis, and atopic dermatitis in children [33]. Di(2-ethylhexyl) phthalate (DEHP), as one of the most common SVOCs, is suspected as a risk factor in childhood asthma and allergic diseases [34,35]. A recent negative consideration in house dust, has been the presence of dibutyl phthalate (DBP), which is thought to have possible associations with previously diagnosed eczema and eye symptoms [36].

In China, an increasing interest in the association between indoor pollution and allergic diseases in children has developed. The China, Child, Homes, Health project conducted a cross-sectional questionnaire survey to examine the associations between in-house environmental factors and the prevalence of asthma and allergies among children aged 3–6 [37]. Deng et al. [38,39] reported that both prenatal and early postnatal exposure to various indoor environmental factors correlated with preschool children suffering from asthma, allergy and various infections. The findings of Liu et al. [40] indicated that culturable fungi and particle concentrations showed strong positive correlations with preschool children’s respiratory health. Mi et al. [41] monitored CO_2_, nitrogen dioxide (NO_2_), ozone (O_3_) and formaldehyde in naturally ventilated schools for pupils (13–14 years old) in Shanghai. Zhan et al. [42,43] investigated indoor NO_2_, CO_2_, sulfur dioxide (SO_2_) and microbial components in settled dust in junior high schools in Taiyuan. However, the above studies: (1) focused mostly on preschool children, and rarely involved children of school age, (2) involved only one or several indoor pollutants, and (3) rarely simultaneously examined these pollutant levels in the home environment and in relation to children’s health.

Schoolchildren aged from 10 to 12 years old are considered to be on the brink of adolescence. Their exposure to such as pollutants is higher than that of adults as their intake is greater per unit body weight via the multiplicity of pathways. Thus, in residential buildings, which are the most likely centres for children’s daily after school activities, chronic air pollutant exposure, in such circumstances as described above, may provide a negative health impact, particularly for the more clinically susceptible. Further data was collected via a systematical investigative study, composed of a questionnaire survey and field investigation related to environmental conditions was then conducted in several major Chinese cities, including Harbin, Dalian [44], Beijing [45], Shanghai [46], Wuhan [47] and Changsha [48]. This study included two stages: (1) cross-sectional questionnaire survey concerning residential characteristics and lifestyles, (2) a detailed field investigation of the environmental conditions of the schoolchildren’s households. The six cities chosen for this study are in different climate zones [49]: Harbin is located in severe cold zone, Beijing and Dalian are located in the cold zone, while Shanghai, Wuhan and Changsha are located in hot summer and cold winter zone.

The objectives of this study were: (1) to inspect environmental conditions in children’s households including building characteristics and lifestyles; (2) to quantitatively assess the levels of indoor pollutants and personal exposure; (3) to compare the differences between the indoor pollution experienced by (a) healthy and (b) unhealthy children’s households and (4) to identify risk factors associated with allergic diseases experienced by children.

## 2. Materials and Methods

### 2.1. Consent

Informed consent was obtained from children’s parents before survey and measurement. All involved families agreed to participate in the survey and part of them allowed us to conduct the measurement in their houses.

### 2.2. QuestionnaireSurvey

During 2012–2013, a questionnaire survey was performed in the 4th- and 5th-grade school pupils. Participants were from the affiliated primary schools of Harbin Institute of Technology, Dalian University of Technology, Beijing University of Technology, Shanghai Jiao Tong University, Huazhong University of Science and Technology, and Hunan University. Approximately 1200 questionnaires were distributed to children’s families in the above mentioned schools. Questionnaires were filled out by the parents of children in accord with their actual situation. Finally, a total of 912 questionnaires were eligible for inclusion in the analysis.

The interview sheet referred to the American Thoracic Society-Division of Lung Disease (ATS-ELD) questionnaires. The contents mainly included [50]: (1) general information of the child; (2) dwelling environment, including residential location, surroundings of household, house size and so on; (3) equipment in everyday use, including heating, cooling, ventilation and the associated degree of humidity, dryness, condensation/mold/visible flow; (4) lifestyle habits, such as feeding style, smoking, the presence of pets, washing frequency of children’s quilts and (5) the specific health status of children and family members and the subsequent allergic symptoms. The questionnaire includes about 80questions and some of them were listed as follows:

“Eczema in the last 2 years”: Has your child had doctor-diagnosed eczema in the last 2 years? (yes vs. no)

“Living near high traffic density roads”: Are you living near high traffic density roads? (yes vs. no)

“Condensation on windows”: Did condensation or moisture occur on the inside or at the bottom of windows(windowpanes) in the child’s bedroom? (yes vs. no)

“Visible mold”: Have you noticed any visible mold on the floor, walls or ceiling in the child’s bedroom? (yes vs. no)

“Visible water flow”: Have you noticed any visible water flow on the floor, walls or ceiling in the child’s bedroom? (yes vs. no)

“Breast feeding”: Has your child been breastfeed for more than 6 months since their birth? (yes vs. no)

“Would you like to cooperate with the detailed measurement about indoor air pollutants in your house? (yes vs. no)”

### 2.3. Field Measurement

Based on the surveyed health conditions, sixty participants (ten children from each city) were selected from the volunteers and detailed field measurements, as indicated above, were conducted in their houses. The sixty children were divided into two groups. Thirty with one or more doctor-diagnosed respiratory and allergic symptoms during the past 2 years, such as wheezing, breathlessness, eczema, urticaria, seasonal or allergic rhinitis, formed Group A. The remaining thirty children who over the last 2 years, had not experienced symptoms listed above, formed Group B.

The field measurements were carried out in the living rooms and bedrooms of each child during the winter of 2013. The measurements taken were as follows: (1) two-week monitoring of air temperature, relative humidity (RH) and CO_2_ level; (2) measurement of PM_2.5_ mass concentration; (3) test of HCHO, acetaldehyde, and total volatile organic compounds (TVOCs) in air; (4) identification of airborne fungi in air and settled fungi in house dust; (5) detection of SVOCs in house dust. Measurements were not taken during “cooking periods” as indoor PM_2.5_ level and carbonyls could be elevated during this period. Additionally, the residents were told not to clean the houses intentionally and keep the indoor environment in a daily state.

#### 2.3.1. Monitoring of Air Temperature, RH and CO_2_ Concentration

The data logger with temperature and humidity sensors (TR-72Ui, T&D Corp., Matsumoto, Japan) was used for monitoring air temperature and RH at 10 min intervals of for 14 days. In each house, the instruments were located at three positions: at heights of 0.1 m and 1.1 m above the floor in the living room, and at a height of 1.1 m above the floor in the child’s bedroom. Care was taken to avoid the direct influence of both solar radiation and indoor heat sources on the sensors. The CO_2_ level in the living room was measured at an interval of 5 min for 14 days. The data logger with CO_2_ sensor (MCH-383SD, Lutron, Taipei, Taiwan) was placed at a suitable location on the floor, to avoid the direct influence of the occupants’ exhalation. 

#### 2.3.2. Monitoring of PM_2.5_ Level

An aerosol monitor (TSI 8354 DustTrak, Shoreview, MN, USA) was applied for measuring PM_2.5_ concentrations in the living room, child’s bedroom and outdoor air on the open balcony. The instrument was installed approximately 1.1 m above the floor to avoid the influence of floor dust and that of human respiration. The PM_2.5_ concentrations were recorded every second of three minutes and the mean values of 180 records were taken as the local PM_2.5_ level.

#### 2.3.3. Sampling and Analysis of HCHO, Acetaldehyde and VOCs

Portable air samplers fitted with a mini pump (SIBATA MP-300, Saitama, Japan) were used to simultaneously sample air at a height of approximately 1.1 m in three areas: above the floor in the living room, the child’s bedroom and outdoors in each selected house. The sampling rate was set to 1 L/min and lasting for 30 min. Air samples for HCHO and acetaldehyde were contained in active gas tubes (SIBATA, DNPH active gas tube), and those for VOCs were collected in solid-phase samplers (SIBATA, charcoal tube standard). As soon as the sampling was completed, the tube was tightly plugged and sealed in an attached bag, specially designed to preserve the active gas tube. HCHO and acetaldehyde were analyzed by the high performance liquid chromatography (HPLC) method proposed by the Japanese Standard Association [51], and VOCs were analyzed by the gas chromatography/mass spectrometry (GC/MS) methods by Jis [52].

#### 2.3.4. Sampling and Analysis of SVOCs in House Dust

House dust for SVOCs detection was gathered within the filter paper (2V, Whatman, Freiburg, Germany) by using a vacuum cleaner (PV-H23, Hitachi, Tokyo, Japan). The house dust samples were collected for 2 min from a 1 m^2^ floor area of the living room and child’s bedroom, and were analyzed by thermal desorption-gas chromatography/mass spectrometry (TD-GC/MS) [52].

#### 2.3.5. Sampling and Analysis of Airborne and Settled Fungi

Airborne fungi were sampled with a one-stage impact or air sampler (BIO SAMP MBS-1000, Midori Anzen Co., Tokyo, Japan) by using the multi-orifice sieve impact method proposed by ISO 16000-18 [53]. Air samples (100 L) from the living room, child’s bedroom and outdoor were blown onto an agar plate containing dichloran-glycerol (DG 18) growth medium. After sampling, the agar plate was immediately covered with its lid and tightly sealed with sealing tape.

A vacuum cleaner (PV-H23, Hitachi Co.) was used to collect house dust samples from both the living room and the child’s bedroom. Dust sampling took place on a 1 m^2^ floor area for 2 min. After passing through a 300 μm mesh, coarse dust particles were removed and the finer dust particles were extracted, weighed and then suspended and agitated in a 10 mL sterilization solution. A 50 μL sample of the resulting suspension was then plated on a DG18 agar plate. The DG18 agar base was incubated at 25 °C for five days. The genera of fungi were determined by morphological analysis according to ISO 16000-17 [54]. The airborne fungi and settled fungi, repectively, were quantified with the number of colonies per cubic meter (CFU/m^3^) and per milligram of dust (CFU/mg).

### 2.4. Statistical Analysis

Multiple logistic regression models were used to evaluate the association between residential factors as presented above and childhood allergic disease, adjusting for gender, age, family history of allergies and environmental tobacco smoke exposure. Associations in the regression analysis were calculated as adjusted odds ratio (OR) with 95% confidence interval (95% CI). A *p* value less than 0.05 was considered statistically significant. The statistical analyses were performed by the IBM SPSS Statistics version 20.0 software (IBM International, Armonk, NY, USA). The Non-parametric Mann-Whitney *U* test was used to compare the differences between two groups of indoor pollutants found in the selected houses. Pearson’s correlation was employed for calculating the correlation coefficients among different air pollutants in houses.

## 3. Results

### 3.1. Questionnaire Data

The prevalence of allergies in children among six cities is shown for comparison in Table 1. Of the 912 children, 46.7% had an allergic symptom in the past, 41% had allergic diseases in the past, 36.4% had current allergies, and 32.6% exhibited evidence of pollinosis and 14% of eczema.

The associations between dwelling environment, lifestyles and childhood allergies were evaluated by multivariate logistic regression models and adjusted for potential confounding covariates including gender (boy vs. girl), age (9 years vs. 10 years vs. 11 years vs. 12 years), family history of allergies (yes vs. no) and environmental tobacco smoke exposure (Table 2). Due to the small number of children suffering from a persistent cough, persistent phlegm and respiratory allergies, multivariate analysis was performed, only, for other allergic diseases.

Based on the above results, childhood pollinosis appears to significantly correlate with such factors as house redecoration activity conducted over the past 10 years, house sizes of less than 60 m^2^, indoor dampness (visible mold, condensation on windows or visible water flow on the floor, walls or ceiling), household pets feeding within the house and light sleep on the part of the child. In addition, living near highly dense traffic roads also possibly increases the risk of allergies in children.

Of interest is that history of allergies and allergic diseases were thought to have a respective correlation with the effects of pets feeding (as mentioned above), and such as low cleaning frequency of quilts. Indoor dampness was recognized as a risk factor for eczema in children. However, of interest is the finding that breast feeding (lasted for more than 6 months since their birth) provides a protective factor for children suffering from pollinosis and current allergies.

### 3.2. Indoor Thermal Environment

**Figure 1:**
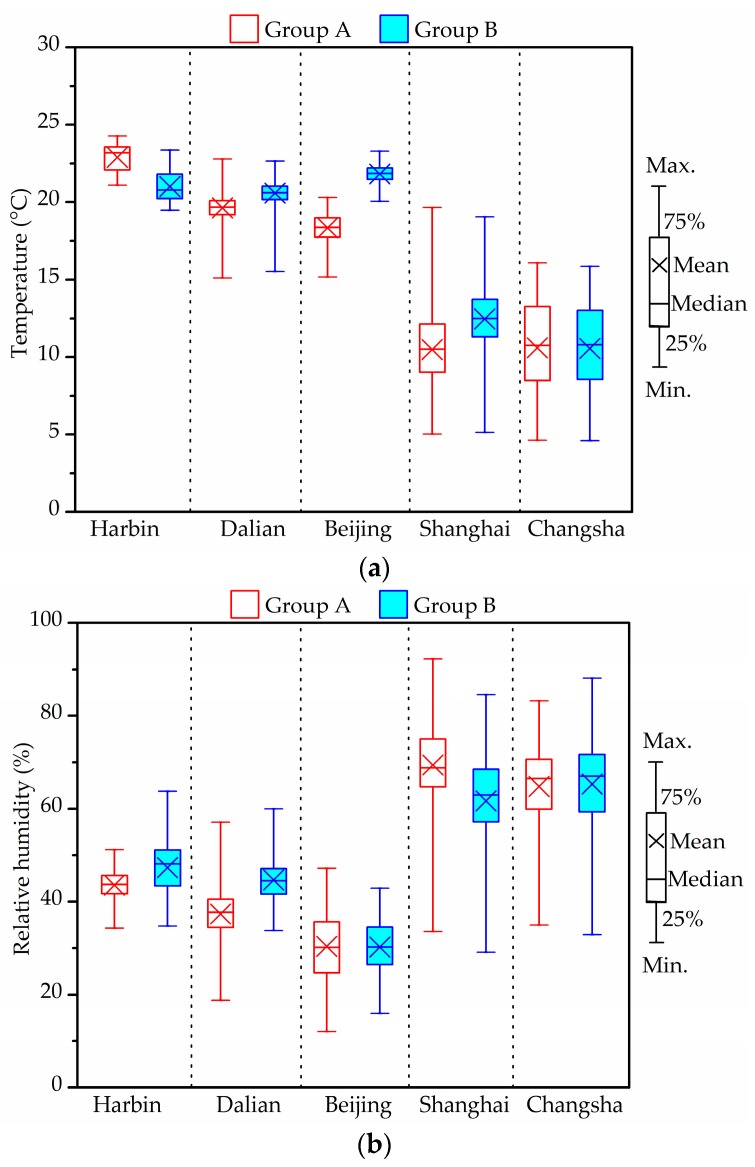
Statistical box plots of indoor temperature (**a**) and relative humidity (RH) (**b**) in two groups in winter.

Figure 1 shows the distributions of the winter indoor temperature and RH experienced by the two groups of children, each group living in different cities. The values were the average value at the three measurement positions as mentioned above.

The characteristics of the thermal environments differ in relation to the individual specified conditions of each measurement group and city. In Harbin, the outdoor temperature varied mainly within the range 1.6 ± 2.4 °C, while RH varied within the range of 65.6 ± 18.2%. Indoor average temperatures, with an average of 22.9 °C in Group A and 21 °C in Group B, due to indoor heating, were much higher than those outdoors. Indoor average RH was less than 50%. Temperature and RH distributions in Dalian and Beijing were similar to those in Harbin. On the whole, the indoor temperature in the investigated houses of the above three cities met the standard value (16–24 °C). Compared with the Chinese indoor air quality (IAQ) standard value (30–60%), indoor RHs in both groups of Beijing and Group A in Dalian were relatively low.

Indoor air temperature in the houses in Shanghai (Group A: 10.5 ± 2.3 °C, Group B: 12.5 ± 2.2 °C) was slightly higher than the outdoor temperature (9.0 ± 3.4 °C). The average temperature in Groups A and B from Changsha were 10.6 ± 2.9 °C (outdoors: 8.3 ± 4.7 °C), thus no difference between the two groups. Indoor RH in the measured houses of these two cities, overall, varied in the range of 60–75%. The respective averages in Groups A/B were 69.4 ± 8.3%/61.7 ± 10.7% in Shanghai and 64.8 ± 9%/65.3 ± 10.7% in Changsha.

Houses in Harbin, Beijing and Dalian were installed with regional central heating, while those in Shanghai, Changsha and Wuhan depended on the use of individual space heaters in winter. However, and of interest, is that no heating systems or devices were in use during the experimental tasks conducted in the Shanghai, Wuhan and Changsha houses.

Yanagi et al. [55] indicated that relative humidity of 70% or more promoted the growth of mold. From the figure, indoor RHs in the Harbin, Dalian and Beijing homes were lower than 50%. Conversely, the percentage of RH exceeding 70% in Groups A and B from Changsha were 32.6% and 36.9%, respectively. In addition, the rates of RH ≥ 70% were higher than 60%, observed more frequently in Group A than in Group B in the Shanghai houses [46]. Dampness and indoor mold appeared to be serious risk factors for allergic symptoms among children.

### 3.3. Indoor CO_2_ Level

Table 3 shows the results of the indoor CO_2_ level tests, conducted in different cities in winter.

From the above results, it is seen that over 32.5% of the measured data in Harbin, Dalian and Beijing are higher than 1000 ppm: factors in excess of which are considered a health risk according to the Chinese IAQ standard [56]. The maximum CO_2_ concentration found in Beijing, was 9754 ppm. The measured CO_2_ in houses in Harbin, Dalian and Beijing was greater than that in Shanghai, Wuhan and Changsha, possibly due to poor natural ventilation, as indicated above. The frequency of CO_2_ concentrations exceeding 1000 ppm in Changsha was in the area of 0.7% in Group A and 1.8% in Group B, hence lower than that in the other two Southern cities studied: Shanghai and Wuhan. As given above, it appears clear that poor household ventilation has the propensity to increase Children’s exposure to and hence impact of chemicals, particles and organisms, thus increasing the risk of such as asthma exacerbations and respiratory infections [57].

### 3.4. PM_2.5_ Level

**Figure 2:**
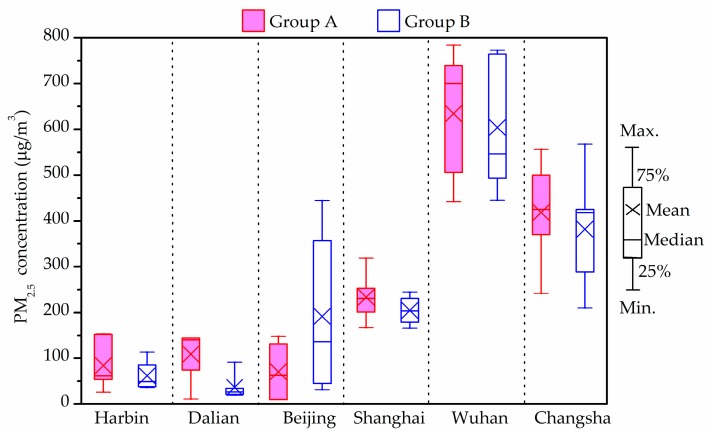
Indoor average fine particulate matter (PM_2.5_) concentrations in winter.

Figure 2 shows the indoor average PM_2.5_ concentrations in the investigated houses in winter. The PM_2.5_ levels in all measured households from Shanghai, Wuhan and Changsha exceeded the upper limit of the Chinese national standard [58], which suggests that the 24-h average PM_2.5_ concentration as 75 μg/m^3^ (Level-2). The indoor average PM_2.5_ concentrations of Group B in Harbin, Dalian and Group A in Beijing were 60 μg/m^3^, 36.5 μg/m^3^ and 71 μg/m^3^, respectively, hence satisfying the suggested limit regarding the protection of health. In addition, the indoor average PM_2.5_ concentrations of Group A except for Beijing conditions, were higher than those of Group B in the other five cities. However, and of interest, this difference showed no statistical significance (*p* > 0.05) between the two groups.

The above statistics show that the PM_2.5_ concentrations in Wuhan and Changsha districts were higher than 500 μg/m^3^. According to the questionnaire responses and field investigation, it was found that some investigated houses were adjacent to roads with dense traffic [48]. Heavy vehicles emissions have been found to release serious PM_2.5_ pollution hence contaminating the adjacent outdoor air. The outdoor air was found to be the major PM_2.5_ source in those rooms with natural ventilation. Thus, combined with the heavy traffic pollution and poor outdoor air quality, the PM_2.5_ levels in indoor rooms could be extremely high in roadside households.

The indoor PM_2.5_ concentrations in Group B in Beijing were higher than those in Group A, however, the average indoor/outdoor (I/O) ratio of PM_2.5_ concentration in Group A (>1) was high than that in Group B (<1). A possible reason was the presence of stronger additional PM_2.5_ sources such as more smokers in Group A houses than that in houses of Group B.

### 3.5. Chemical Compound Concentration (HCHO, Acetaldehyde and TVOCs)

**Figure 3:**
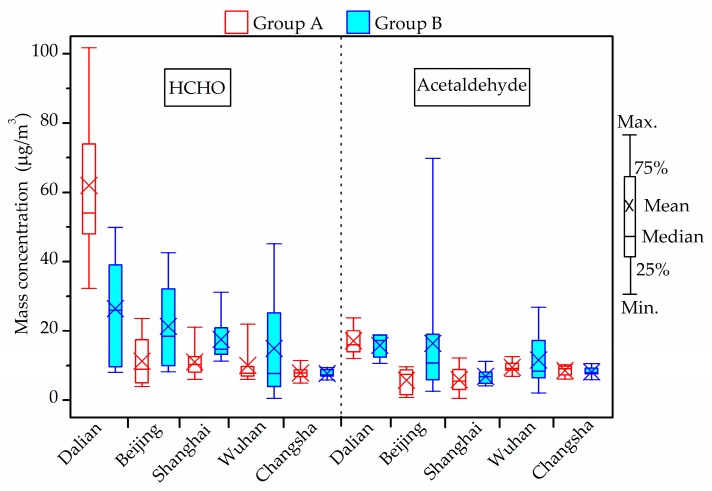
Indoor formaldehyde (HCHO) and acetaldehyde concentrations in winter.

Figure 3 shows the concentrations of indoor chemical compounds, including HCHO and acetaldehyde in winter. The maximum HCHO concentration in all investigated houses was generally lower than the current guideline (100 μg/m^3^) provided by the Chinese national standards [56] except in one living room in Dalian (101.7 μg/m^3^). The highest concentration in Beijing, Shanghai, Wuhan and Changsha was 42.6 μg/m^3^, 31.1 μg/m^3^ [46], 45.2 μg/m^3^ [47] and 11.4 μg/m^3^, respectively, lower than half of the limit.

As for acetaldehyde, the current indoor air quality standards for residential buildings in China do not involve any reference limit. According to the Japanese national standard which suggests the upper acetaldehyde level of 48 μg/m^3^ [59], the maximum acetaldehyde concentration in all investigated houses was generally lower than this guideline, except in one living room in the houses of Beijing (69.8 μg/m^3^) [45]. Acetaldehyde levels in the measured houses of other cities varied from 0.5 to 26.9 μg/m^3^.

**Figure 4:**
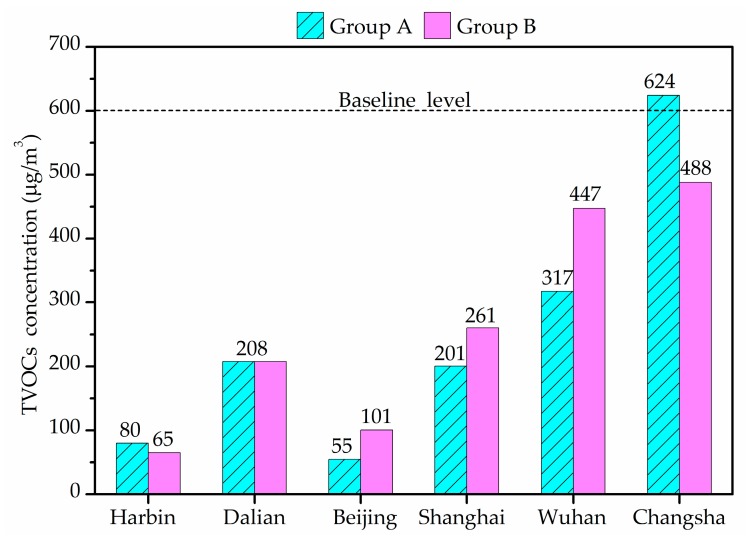
Indoor average total volatile organic compounds (TVOCs) concentrations in winter.

Figure 4 shows the indoor average TVOCs concentrations in winter. TVOCs concentrations revealed large differences between different cities houses. The average of TVOCs in Harbin and Beijing were less than 100 μg/m^3^ and those in Dalian, Shanghai and Wuhan were less than 600 μg/m^3^, the limit stipulated by the Chinese national standards [56]. However, the average indoor TVOCs concentration of Group A in Changsha was 624 μg/m^3^, exceeding the reference value. TVOCs concentration in Wuhan varied in a wide range and exceeded 600 μg/m^3^ in five air samples. There was no significant difference between Groups A and B.

### 3.6. SVOCs Concentrations of Household Dust on Floors

**Figure 5:**
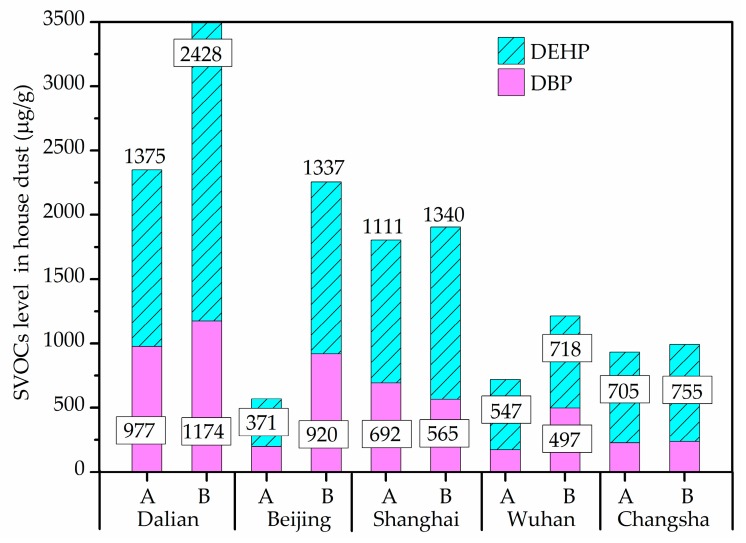
Semi-volatile organic compounds (SVOCs) level in house dust in winter.

The most frequently detected components of SVOCs in house dust were found to be DEHP and DBP, with DEHP, alone, was present in all dust samples. Figure 5 shows the DEHP and DBP levels in all dust samples from different cities. In the revised standard Restriction of Hazardous Substances (RoHS) 2.0 (2015/863/EU) officially implemented in 2019 [60], DEHP and DBP, with the limit value of 1000 μg/g, were added. Compared with this guideline, DEHP and DBP in about 32.3% of the dust samples exceeded 1000 μg/g, with maximum concentrations detected in Dalian houses. However, the difference of DEHP and DBP between the two groups showed no statistical significance.

### 3.7. Fungi

**Figure 6:**
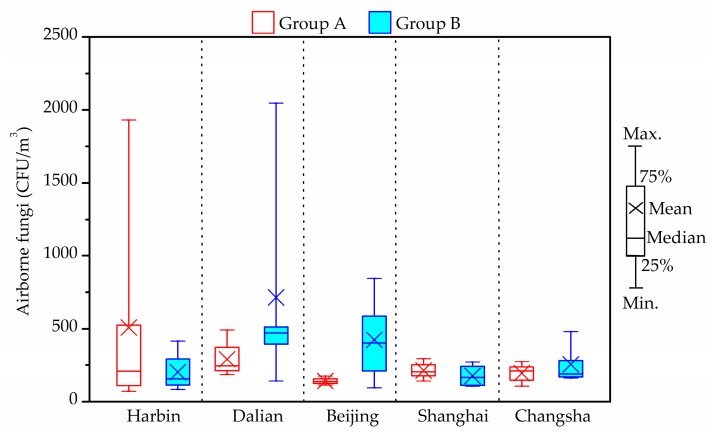
Indoor average level of airborne fungi in air in winter.

**Figure 7:**
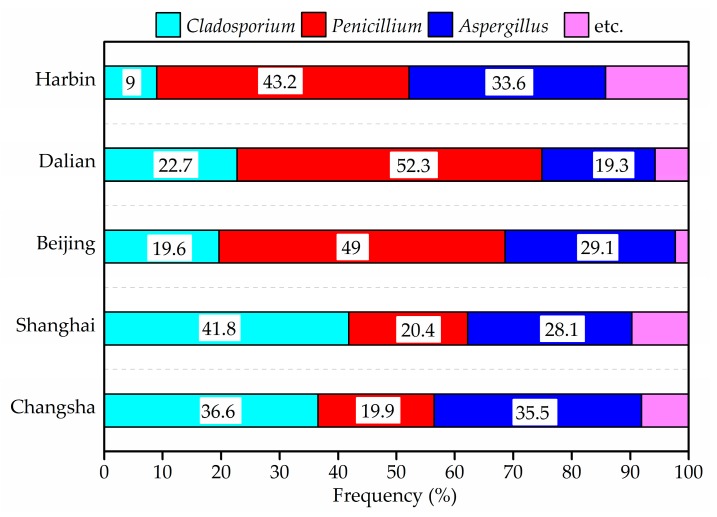
Frequency of airborne fungi species in indoor air in winter.

**Figure 8:**
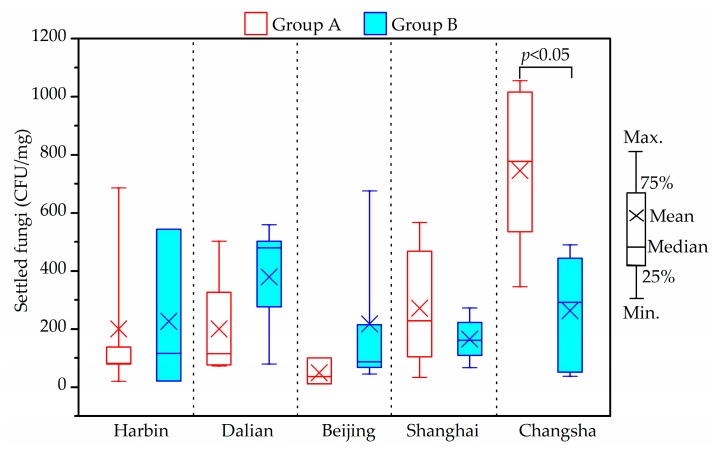
Indoor average level of settled fungi in house dust in winter.

**Figure 9:**
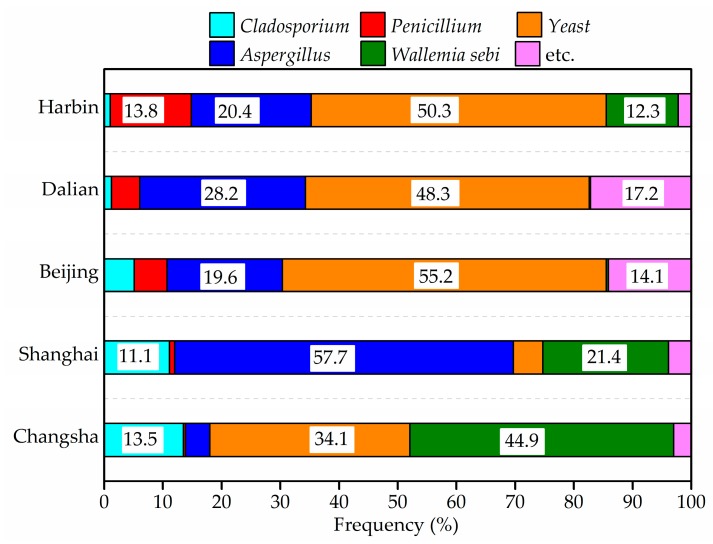
Frequency of settled fungi species in house dust in winter.

The results concerning the airborne fungi in the air and settled fungi in house dust are presented in Figure 6, Figure 7, Figure 8 and Figure 9. Three species of fungal allergen, i.e., *Cladosporium*, *Aspergillus* and *Penicillium* were the dominant species in the air and generally detected in all air samples. They accounted for 23.5%, 27.9% and 40.8%, respectively of indoor airborne fungi. As shown in Figure 7, the proportion of the main fungi species in air samples varied greatly from city to city. *Penicillium* in air samples from Harbin, Dalian and Beijing accounted for more than 43% of indoor airborne fungi, significantly higher than that of other species. Conversely, the percentage of *Cladosporium* in air samples from Shanghai and Changsha showed an increase. 

The total bacterial count in all air samples was fond to be always below the upper limit of the 2500 CFU/m^3^ proposed by the Chinese national standards [56]. A stricter limit of 1000 CFU/m^3^ for airborne fungal spores in indoor air was suggested by the Architectural Institute of Japan [59]. Compared with this guideline, the indoor average airborne fungi level in one house in Group A from Harbin (1930 CFU/m^3^) and one house in Group B from Dalian (2048 CFU/m^3^) exceeded the limit. The airborne fungal levels in indoor air from Shanghai and Changsha samples were less than 500 CFU/m^3^. The maximum concentrations of indoor fungal found in Beijing was 845 CFU/m^3^, and detected in a house of Group B. However, there were no obvious differences between two groups (*p* > 0.05).

As shown in Figure 8 and Figure 9, yeast, *Aspergillus*, *Wallemiasebi*, *Cladosporium* and *Penicillium* were more frequently detected in house dust than other fungal species. The five dominant species accounted for around 92% of settled fungi in house dust. Some dust samples from Harbin, Dalian and Beijing were found to have no detectable fungi, due to the fewer samples that were taken.

The maximum indoor average settled fungi level was 1016 CFU/mg, and found in Changsha samples. The difference between Groups A and B for settled fungi from Changsha houses was significant (*p* < 0.05). However, no specific tendency, in this respect, was observed in the two groups in the other four cities (*p* > 0.05).

## 4. Discussion

### 4.1. Children’s Exposure to PM_2.5_, HCHO, Acetaldehyde and TVOCs

As regards this current study, human exposure pathways to air pollutants mainly include inhalation, oral ingestion and dermal absorption indoors [61]. Respiratory inhalation is the main route for PM_2.5_, HCHO, acetaldehyde and TVOCs. To evaluate children’s exposure to indoor environmental pollution, noting the time children spent in households was necessary if an accurate assessment record was to be achieved. According to the questionnaire responses in the six cities, the average daily time spent in living rooms and child’s bedroom was 4 h and 10 h, respectively. Personal inhalation exposure could be estimated by the methodology proposed by the United States Environmental Protection Agency (US EPA) [62]. The formula is described as Equation (1):

(1) $$E_{inhalation}=C_{j}\times IR_{i}\times t_{ij}$$

where *C_j_* relates to the concentration of air pollutants measured in this study (μg/m^3^); *IR* relates to the inhalation rate of children (m^3^/h), in accordance with the recommended value by EPA [63]; *t* relates to the indoor exposure time (h), while *j* is the microenvironment room. Children’s daily exposures to pollutants, as indicated above, were given in detail in Table 4.

In most of the houses investigated, a child’s daily dose of air pollutants in the bedroom was significantly higher than that in the living room (*p* < 0.05), due to the longer time spent by the child in the bedroom over one day. The total daily dose of air pollutants which included PM_2.5_, HCHO, acetaldehyde and TVOCs in Groups A and B varied in different cities. In the Dalian and Changsha houses, the total daily doses of Group A, (namely Dalian: 3594 μg/day, Changsha: 7835 μg/day), were higher than those in Group B, (namely Dalian: 2467 μg/day, Changsha: 6855 μg/day). However, the opposite results were found in the Beijing, Shanghai and Wuhan houses.

### 4.2. Children’s Exposure to DBP and DEHP in House Dust

Children are exposed to DBP and DEHP from house dust through ingestion and dermal absorption [63,64,65]. Their daily intakes were estimated through Equations (2) and (3) as follows:

(2) $$E_{ingestion}=\frac{C_{dust}\times f_{1}\times f_{2}}{M}$$

where $C_{dust}$ is the DBP and DEHP level in house dust detected in the study (μg/g); $f_{1}$ is the percentage of time spent indoors over a day; $f_{2}$ is the dust ingestion rate, 0.05 g/day [64]; *M* is the body weight of children, as indicated in [66]:

(3) $$E_{dermal}=\frac{C_{dust}\times f_{1}\times A\times m\times f_{3}}{M}$$

where *A* is the body surface area (cm^2^/day). According to [64], *A* is 3067 cm^2^/day for children aged 9–10 years and 3692 cm^2^/day for children aged 11–12 years; *m* is the dust which adheres to skin, 0.096 mg/cm^2^ [64]; *f*_3_ is the fraction of DBP and DEHP absorbed in the skin, 0.001556 of DBP and 0.000106 of DEHP [64]. Table 5 presents a summary of the children’s daily doses of DBP and DEHP based on house dust present in different cities.

Children’s daily intake of DBP and DEHP from house dust in child’s bedrooms were significantly higher than such intakes from living rooms (Mann-Whitney *U* test, *p* < 0.05). However, the Pearson correlation coefficient (*r*) of daily exposure to DBP and DEHP betweenthe child’s bedroom and the living room showed that there was no significant correlations in the different rooms (*r* = 0.48, *p* > 0.05). The daily intakes of DBP and DEHP from house dust via ingestion varied from 638 to 2974 ng/day-kw/day, with an average of 1407 ng/day-kw/day, while 1.7 to 11.1 ng/day-kw/day with an average of 5.5 ng/day-kw/day through dermal absorption. Children in the houses from Dalian, Beijing and Shanghai ingested more phthalates from house dust than children from Wuhan and Changsha, with statistical significance (*p* < 0.05).

### 4.3. Relationships among Environmental Pollutants

In order to ascertain the possible relevance of measured environmental pollutants, Person correlation coefficients were calculated and are presented in Table 6. Concerning the correlations between pollutants in the living room (L) and the child’s bedroom (C), PM_2.5_, HCHO, acetaldehyde, settled fungi and DEHP presented strongly positive correlations (*r* > 0.85, *p* < 0.01), while TVOCs and DBP showed moderate correlations (*r* > 0.7, *p* < 0.05). It was quite clear that PM_2.5_ in particular, significant correlations were found between living rooms and child’s bedrooms, in five cities apart from Shanghai. In addition, indoor PM_2.5_ concentrations were significantly correlated with outdoors (*r* > 0.9, *p* < 0.01), thus suggesting that outdoor PM_2.5_ pollution has a strong influence on indoor PM_2.5_ pollution.

From the above results, a correlation between living rooms and child’s bedrooms of airborne fungi was shown (*p* < 0.05). Meanwhile, airborne fungi were related to PM_2.5_ and settled fungi associated with DBP and DEHP. The correlations among different air pollutants were all statistically significant (*p* < 0.05). These relationships indicated that the associated compounds might come from the same indoor sources. Therefore, even if the levels of these mentioned pollutants did not exceed the upper limit, and the difference between the two groups of children’s exposures to these air pollutants was not significant, the combined influence of these pollutants could have adverse effects on the health of children. As a result, children’s health could be affected by the synthetic action of air pollutants such as PM_2.5_ and fungi.

### 4.4. Comparison of Environmental Conditions in Houses among Different Cities

This current study has examined indoor thermal and environmental conditions and their health risk to children in six Chinese cities. The environmental parameters in different cities displayed both similarities and differences. In terms of similarities, indoor levels of HCHO, acetaldehyde were practically lower than the guideline except for HCHO concentration in one investigated Dalian house as was acetaldehyde concentration in one measured Beijing house. Indoor average TVOCs levels were lower than 600 μg/m^3^ except in the selected Changsha houses.

The most frequently measured compounds in house dust in each city were DBP and DEHP. Lastly, the most frequently detected airborne fungi in the air were *Cladosporium*, *Aspergillus* and *Penicillium*. The differences between the different cities are given below:

(1) As indoor heating equipment is generally used in winter in Northern cities such as Harbin, Dalian and Beijing, the indoor temperature in the houses varied in the range of 15–24 °C, and indoor average RH was less than 50%. Indoor RH in Beijing was relatively low, indoor RH in Shanghai and Changsha varied in the range of 60–75%. Whereas, the indoor RH percentage exceeding 70% in Changsha was approximately 33%, however, the rates of indoor RH exceeding 70% in Shanghai were more than 60%.
(2) The CO_2_ levels in houses of Harbin, Dalian and Beijing were much higher than that in Shanghai, Wuhan and Changsha, as a consequence of poor natural ventilation caused by the use of indoor heating and low outdoor temperature.
(3) The PM_2.5_ level in the investigated houses of Wuhan and Changsha were far higher than the upper limit. PM_2.5_ concentrations in other investigated cities were relatively low.
(4) According to the Japanese standard, indoor airborne fungal levels in some investigated houses from Harbin and Dalian exceeded the limit of 1000 CFU/m^3^. The growth and reproduction of fungi flourish in a particular environment such as with a temperature range of 20–35 °C and a relative humidity range of 75–100% [67]. The indoor temperature in Harbin and Dalian were conducive to the growth and propagation of airborne fungi. Thus, the schoolchildren in Harbin and Dalian were naturally exposed to greater fungal pollution than those in Beijing, Shanghai and Changsha.
(5) No obvious difference between settled fungi in Groups A and B in Harbin, Dalian, Beijing and Shanghai was found. In contrast, a significant difference was observed between the two groups in Changsha.

### 4.5. Limitations

There are some limitations in this study. The first potential limitation is the recall bias. The questionnaires were answered by children’s parents retrospectively. Generally, parents with family allergic history or whose child had been doctor-diagnosed with allergic diseases are likely to remember their children’s allergic symptoms and the related indoor environmental factors as well. However, some recall bias is inevitable. Secondly, in this study we only measured the pollutions of PM_2.5_, HCHO, TVOCs, SVOCs and fungi. It is important to further investigate the roles of the other air pollutants such as SO_2_ and NO_2_. Finally, the measurements did not take place over a full year, thus the indoor pollution conditions in other seasons is still unknown. Therefore, more detailed research work is needed in future.

## 5. Conclusions

A systematic study supported by a questionnaire survey and field investigation has been conducted. The focus areas were the following six typical cities in China: Harbin, Dalian, Beijing, Shanghai, Wuhan and Changsha. Field investigations focused on a selection of 10 houses in each city. Of prime interest was the children’s residential thermal environment and the identification of the extent and types of indoor primary pollutants in winter. Conclusions are drawn as follows:

(1) Living environment factors such as homes near high traffic density roads, redecoration, small house size, dampness and lifestyles such as pets feeding within the family living space significantly contributed to allergic symptoms in children. Breast feeding (>6 months) was a protective factor for children suffering from pollen hypersensitivity and diagnosed allergies.
(2) Indoor RHs in Group A (unhealthy children) of Dalian, and in many of those houses investigated in Groups A and B (healthy children) in Beijing were below 30%. The ratio of indoor RH exceeding 70% in Changsha and Shanghai were respectively about 35% and 60%. The above inappropriate thermal environments represent potential health risk factors as regards children’s health.
(3) Much of the CO_2_ measured data exceeded Chinese limit level (1000 ppm) in different cities, possibly revealing that poor ventilation in houses could directly affect childhood health. The children in Group A in the investigated cities, except for Beijing were exposed to higher indoor PM_2.5_ levels than those in Group B according to Chinese national standard suggesting PM_2.5_ could be considered a risk factor for respiratory and allergic symptoms in children.
(4) Except for one house in Dalian, HCHO concentration in all the other investigated houses did not exceed the upper limit of Chinese IAQ standard. Similar results were also observed for acetaldehyde levels, only one house in Beijing exceeded the guideline according to the Japanese national standard. Children in Group A in Changsha were exposed to higher TVOCs levels in their houses than those in Group B. Due to the synthetic action of these chemical compounds, it is reasonable to take them as important and prevalent indoor pollutants for the children, especially in child’s bedroom where children spent more time.
(5) DBP and DEHP were the most frequently detected components of SVOCs in house dust. SVOC levels higher than 1000 μg/g (European Union standard) should be noted as potentially having adverse health effects.
(6) *Cladosporium*, *Aspergillus* and *Penicillium* were simultaneously detected in indoor air and house dust, which could be suspected as adverse factors for children allergies.

These findings are helpful to evaluate and design healthy indoor living environment for schoolchildren, and provide some reference for future studies on assessing housing environmental risks for childhood health in China. Certainly, further research is needed to better understand the potential risk factors and the effect of indoor pollutants in other seasons of the year.

## Figures and Tables

**Table 1:** The prevalence of childhood allergies and symptoms among six Chinese cities.

Symptoms	Prevalence (%)						
	Total	Harbin	Dalian	Beijing	Shanghai	Wuhan	Changsha
	(*n* = 912)	(*n* = 127)	(*n* = 120)	(*n* = 150)	(*n* = 205)	(*n* = 170)	(*n* = 140)
Persistent cough	2.2	2.36	0.8	2	2	2.4	3.6
	(*n* = 20)	(*n* = 3)	(*n* = 1)	(*n* = 3)	(*n* = 4)	(*n* = 4)	(*n* = 5)
Persistent phlegm	2	2.36	1.7	2	2	2.4	1.4
	(*n* = 18)	(*n* = 3)	(*n* = 2)	(*n* = 3)	(*n* = 4)	(*n* = 4)	(*n* = 2)
Respiratory allergy	8.8	5.51	9.2	0.7	12.2	12.9	10
	(*n* = 80)	(*n* = 7)	(*n* = 11)	(*n* = 1)	(*n* = 25)	(*n* = 22)	(*n* = 14)
Pollinosis	32.6	29.13	32.5	22	37.6	36.5	35
	(*n* = 297)	(*n* = 37)	(*n* = 39)	(*n* = 33)	(*n* = 77)	(*n* = 62)	(*n* = 49)
Past allergic diseases	41	43.31	38.3	28	50.7	43.5	37.9
	(*n* = 374)	(*n* = 55)	(*n* = 46)	(*n* = 42)	(*n* = 104)	(*n* = 74)	(*n* = 53)
Current allergies	36.4	32.28	35.8	25.3	42.9	39.4	38.6
	(*n* = 332)	(*n* = 41)	(*n* = 43)	(*n* = 38)	(*n* = 88)	(*n* = 67)	(*n* = 54)
Past allergies	46.7	43.31	55	41.3	50.7	50.6	37.9
	(*n* = 426)	(*n* = 55)	(*n* = 66)	(*n* = 62)	(*n* = 104)	(*n* = 86)	(*n* = 53)
Eczema	14	11.02	10.8	10.7	19	16.5	12.9
	(*n* = 128)	(*n* = 14)	(*n* = 13)	(*n* = 16)	(*n* = 39)	(*n* = 28)	(*n* = 18)

**Table 2:** Multivariate logistic regression analysis of the dwelling environment, lifestyles and childhood allergic diseases in terms of adjusted odds ratio (OR) with 95% confidence interval (95% CI).

Factors	Pollinosis *n* (OR, CI)	Current Allergies *n* (OR, CI)	Past Allergies *n* (OR, CI)	Past Allergic Diseases *n* (OR, CI)	Eczema *n* (OR, CI)
Living near high traffic density roads	-	1.61	-	-	-
		(2.36, 5.41) *			
House redecoration	3.52	3. 34	-	-	-
	(1.55, 7.97) *	(1.6, 6.83) *			
House size (<60 m^2^)	2.31	-	-	-	-
	(1.29, 4.13) *				
Dampness	4.31	-	-	-	4.07
	(2.34, 7.64) *				(1.96, 8.48) *
Household pets	3.39	-	4.42	-	-
	(1.22, 9.27) *		(1.59–12.27) *		
Light sleep	1.61	-	-	-	-
	(5.39, 11.28) *				
Breast feeding	0.21	0.19	-	-	-
	(0.1–0.43) **	(0.1–0.42) **			
Low cleaningfrequency of quilts	-	-	-	2.08	-
				(1.15, 3.77) *	

* denotes *p* < 0.05, ** denotes *p* < 0.01.

**Table 3:** Indoor CO_2_ concentration in different cities in winter.

Item	Groups	Harbin	Dalian	Beijing	Shanghai	Wuhan	Changsha
Average value (ppm)	A	945	926	982	758	689	479
	B	1098	1086	1227	701	808	496
Range (ppm)	A	313–3083	333–3595	347–9540	327–2480	330–2158	316–1806
	B	324–2975	279–6588	237–9794	306–3200	312–2970	294–2029
The ratio exceeding 1000 ppm	A	43.4%	34%	40.8%	17.1%	10%	0.7%
	B	55.1%	32.5%	43.5%	14.1%	38.2%	1.8%

**Table 4:** Child’s daily exposure to PM_2.5_, HCHO, acetaldehyde and TVOCs in the investigated households (μg/day).

Item	Room	Dalian		Beijing		Shanghai		Wuhan		Changsha	
		A	B	A	B	A	B	A	B	A	B
PM_2.5_	L	239	96	107	727	392	368	1501	1324	491	687
	C	699	194	340	1469	1249	1043	2490	3234	2831	2217
HCHO	L	220	54	23	48	17	28	17	30	9	15
	C	193	190	95	82	70	92	67	89	54	45
Acetal-dehyde	L	52	39	13	52	9	12	19	23	10	16
	C	83	97	55	50	38	36	57	69	62	47
TVOCs	L	698	770	102	225	309	166	386	726	808	993
	C	1411	1027	272	312	1170	2395	1112	1764	3570	2834
Total		3594	2467	1008	2965	3255	4140	5649	7253	7835	6855

L: living room; C: child’s bedroom; A, B: Groups A and B.

**Table 5:** Child’s daily exposure to DBP and DEHP in the investigated households in winter (ng/day-kw/day).

Room	Exposure Routes	Dalian		Beijing		Shanghai		Wuhan		Changsha	
		A	B	A	B	A	B	A	B	A	B
L	Ingestion	448	580	211	347	478	708	92	318	110	167
	Dermal absorption	1.5	1.7	0.6	2.0	1.9	2.3	0.5	1.6	0.3	0.6
C	Ingestion	1866	2394	1178	1640	789	752	546	405	450	591
	Dermal absorption	9.1	9.4	3.8	8.8	3	2.1	1.2	1.7	1.5	1.6
Total		2325	2985	1393	1998	1272	1464	640	726	561	761

L: living room; C: child’s bedroom; A, B: Groups A and B.

**Table 6:** Pearson correlation coefficients for household environmental pollutants in six cities.

Sites	Environmental Pollutants	Other Environmental Pollutants	Correlation Coefficient (*r*)	Sig. (2-Tailed) (*p*)
Harbin	PM_2.5_ (L)	PM_2.5_ (C)	0.946 **	0.004
	Airborne fungi (L)	Airborne fungi (C)	0.998 **	0.000
Dalian	PM_2.5_ (L)	PM_2.5_ (C)	0.905 **	0.000
	Airborne fungi (L)	Airborne fungi (C)	0.971 **	0.000
	Settled fungi (L)	DBP (L)	0.689 *	0.04
	Settled fungi (L)	DEHP (L)	0.72 *	0.029
Beijing [45]	PM_2.5_ (L)	PM_2.5_ (C)	0.98 **	0.000
	PM_2.5_ (L)	PM_2.5_ (O)	0.862 **	0.001
	PM_2.5_ (C)	PM_2.5_ (O)	0.874 **	0.001
	HCHO (L)	Acetaldehyde (L)	0.801 **	0.004
	HCHO (C)	Acetaldehyde (C)	0.775 **	0.007
	Airborne fungi (L)	Airborne fungi (C)	0.886 **	0.000
	Airborne fungi (C)	PM_2.5_ (C)	0.892 **	0.000
	Settled fungi (L)	Settled fungi (C)	0.974 **	0.000
	Settled fungi (C)	DEHP (C)	0.758 *	0.042
	Settled fungi (C)	DBP (C)	0.805 *	0.034
	DEHP (L)	DEHP (C)	0.873 **	0.000
Shanghai [46]	HCHO (L)	HCHO (C)	0.833 **	0.003
	Acetaldehyde (L)	Acetaldehyde (C)	0.904 **	0.000
	Airborne fungi (L)	Airborne fungi (C)	0.76 *	0.011
	Settled fungi (L)	PM_2.5_ (L)	0.660 *	0.038
Wuhan	PM_2.5_ (L)	PM_2.5_(C)	0.965 **	0.002
	PM_2.5_ (L)	PM_2.5_ (O)	0.927 **	0.008
	PM_2.5_ (C)	PM_2.5_ (O)	0.879 *	0.021
	HCHO (L)	HCHO (C)	0.879 **	0.009
	Acetaldehyde (L)	Acetaldehyde (C)	0.946 **	0.001
	HCHO (L)	Acetaldehyde (L)	0.982 **	0.000
	HCHO (C)	Acetaldehyde (C)	0.96 **	0.001
	DBP (L)	DBP (C)	0.778 *	0.039
Changsha [48]	PM_2.5_ (L)	PM_2.5_ (C)	0.958 **	0.000
	PM_2.5_ (L)	PM_2.5_ (O)	0.977 **	0.000
	PM_2.5_ (C)	PM_2.5_ (O)	0.921 **	0.000
	TVOCs (L)	TVOCs (C)	0.746 *	0.013

L: living room; C: child’s bedroom; O: outdoor. * *p* < 0.05; ** *p* < 0.01.
